# Supplementary material for: Memories in a network with excitatory and inhibitory plasticity are encoded in the spiking irregularity
Source: PLoS Comput Biol. 2021 Nov 11;17(11):e1009593. doi: 10.1371/journal.pcbi.1009593 (PMC8610285; doi:10.1371/journal.pcbi.1009593)
Supplement: Attachment — Submitted filename: response_reviewers.pdf [file pcbi.1009593.s011.pdf]

Dear Editors,

Thank you very much for your consideration. We have performed a couple of new analysis and updated the manuscript according to the Reviewers' suggestions. Please find below a point-by-point answer to their comments.

We would like to thank the reviewers for their very interesting feedback. We believe their suggestions have made the manuscript significantly better.

Please note that the line numbers in our answers refer to the manuscript with highlighted changes.

> *Reviewer #1: This paper explores how memories can be encoded in the spiking irregularity of "silent" cell assemblies -- that is, cell assemblies with low mean firing rates. Quiescence in such assemblies is maintained through EI balance, and it has traditionally been thought that memories could be retrieved from these assemblies by momentarily disrupting this balance, either through excitation or disinhibition from an external input. This paper proposes the novel idea that silent assemblies can also encode for memories through the irregularity of their spiking pattern rather than just the firing rates alone. Indeed, this irregularity could allow downstream neurons to read out the memory without needing to disrupt the EI balance, making such a mechanism energetically more efficient.*
>
> *The authors show that after an assembly is momentarily stimulated by external inputs, EI balance will help maintain the assembly's mean firing rate at the same low level as the background population (hence their "silence"), but the assembly neurons' spiking will be more irregular compared to their non-assembly counterparts (irregularity is quantified by the coefficient of variance or CV). This mechanism depends on both excitatory and inhibitory plasticity, both to encode the new memory and to maintain EI balance after encoding. In single neuron simulations, they show that different inputs into a single neuron will lead to different levels of irregularity but the same mean firing rate, suggesting that spiking irregularity can serve as a mechanism for coding different inputs. They also show how short-term plasticity offers a mechanism for decoding the level of irregularity in assemblies. And they finish by exploring how spiking irregularity can contribute to the longevity of memories.*
>
> *Overall, this paper is clearly written and easy to follow. The main ideas are well-supported by a series of simulations with LIF neurons that follow a logical progression. And while this reviewer is not an expert on the memory literature, I did find both the context of this work and how their contribution fits in with previous work on the subject well explained. Indeed, I think the clarity is a strength and would help this paper reach a wider audience. I do have a few suggestions for how this paper could be further strengthened:*
>
> ** in the simulation for Fig 2, the authors compared a simulation with fixed synaptic weights against one with plastic weights. The fixed weights (W_{E->E}) were set to J. I am wondering what range of values (e.g. the mean and variance) did the plastic weights cover. Was this significantly different from J? If so, it may be helpful if the authors also ran a simulation*

where the fixed weights were better matched at least to the mean value of the variable weights.

That is an interesting observation. To answer this question, we calculated the mean synaptic weight for the different values of CV of the plastic simulations for Figure 2. We found that the plastic weights had a mean approximately equal to J for the simulation with CV=0.4 (Supplementary Figure 1A left). Of course, the mean plastic weight increased for the simulations with larger CV (see Supplementary Figure 1A right, for the synaptic weight distribution when CV=1.4). We have therefore rerun the simulations with static weights (as in Figure 2) using the mean synaptic weight of the plastic simulations with CV=0.4 instead of J. We have also repeated the same simulation using the mean synaptic weight of the simulations with CV=1.4 and included it in the Supplement (Supplementary Figure 1B-G).

** in the network simulations, the authors only gave one measurement for the assembly CV and one measurement for the readout firing rate. To read out the spiking irregularity of a neuron, a downstream neuron would presumably need to integrate input spikes across a time window. To get a sense of how quickly a downstream neuron can reliably measure the irregularity of an upstream neuron, it would be helpful to plot time courses of the CVs as well as of the readout firing rates, preferably on the same time axis.*

This is also a very interesting point. We have plotted the time series of CV (using time bins of 1 second) as well as the time series of the readout firing rates (Supplementary Figure 4), as suggested by the reviewer. Please note, however, that estimating the CV from small time bins could lead to an overall underestimation of the CV values. This is because the ISI distribution is skewed, and, by sampling a small number of intervals, there is an under-representation of large intervals. Using larger time bins (e.g. 50s), on the other hand, would imply estimating the CV from a non-stationary process. Indeed, after the assembly stimulation, the CV is changing at a faster time scale than 50s due to changes in the excitatory and inhibitory weights, as well as changes in assembly neurons own firing rate (Supplementary Figure 4A-C).

Note that the firing rate of the readout neuron is a function of both mean firing rate and CV of the assembly neurons. Following assembly stimulation, the firing rate of assembly neurons is high, and their CV is low, which results in a high firing rate of the readout neuron. A high firing rate of assembly neurons triggers the increase of inhibitory weights which will eventually lead to assembly neurons firing again at target rate, but with elevated CV. To isolate the effect of CV alone, we performed a new simulation (Supplementary Figure 2), where we generated spike trains with a constant firing rate but changing CVs and recorded the read-out neurons.

** similarly, in Fig 4, the authors only plotted the time course of the weights. Since memory is manifested as spiking irregularity, it would also be informative to plot the time course of the CV -- this would provide a more direct measurement of a memory's effective decay rate, since downstream neurons have access only to this information and not to assemblies' synaptic strengths.*

We have plotted the time course of CV using bins of 1s (Supplementary Figure 9), as suggested by the reviewer.

** in line 140, the authors commented on how assembly neurons were more correlated and pointed to the raster plot of Fig 3G as evidence. Rather than just relying qualitatively on raster plots, I think it'd be better if the authors could provide a more quantitative measure of correlation here. One option is the Pearson correlation that the authors used in Fig 4, but I think temporal cross-correlations can also be informative, as this would give a better sense of how correlated spike timings are. For this, the authors can consider three types of cross-correlations: average cross-correlations between assembly neurons, average cross-correlations between an assembly neuron and a non-assembly neuron, and average cross-correlations between non-assembly neurons.*

We thank the reviewer for the nice suggestion; we have added the cross-correlations instead of distribution of correlation coefficients (Supplementary Figure 6).

** in lines 149-150, the authors speculate that the larger firing rate of the assembly's readout neuron is due to both the assembly's higher CV as well as higher correlations. The speculation about the correlations is reasonable, and there is a simple test the authors could perform to help validate this. The authors can introduce a random lag to each assembly neuron's spike train to break up the correlations, and then feed these lagged spike trains into the readout neuron. A lower firing rate would support the hypothesis that correlations do contribute to the higher firing rate.*

We thank again the reviewer for this great suggestion. We performed the proposed analysis (Supplementary Figure 7): Shifting the spike trains led to a lower response of the readout neuron, as predicted. However, it actually led to a zero rate response of the readout neuron, which raised a new question of whether there was any effect of the higher CV of assembly neurons on the readout response.

In order to test this, we repeated the proposed analysis while increasing the constant input current the readout neuron received from 150pA (Figure 2, Figure 3, Supplementary Figure 7A) to 180pA (Supplementary Figure 7B). When the readout neuron received a higher constant input current, shifting the spike trains led to an overall smaller readout response. However, the readout neuron still responded with a higher rate to neurons within the assembly than to neurons outside the assembly (Supplementary Figure 7B), indicating that higher CV can indeed lead to higher readout response.

** in the Discussion, it could be helpful if the authors would comment on the fidelity of memory retrieval with their set-up. While the authors did show in a simple example how different coefficients of variability can lead to different firing rates in readout neurons, it is not immediately clear how this would scale to the encoding and decoding of more complex inputs. Specifically, two questions come to mind. Can different inputs get encoded with similar patterns such that an STP-based readout would have difficulty distinguishing one memory from the other? And as the synpatic weights decay, would the original memory start resembling other memories? While I appreciate that a thorough investigation of these questions is a subject for future work, it'd be nice to have some preliminary discussion of it in the paper.*

In our current work, we assumed that the connections from the assemblies to the readout neurons had already been learned and therefore we hard-wired the assemblies to the readout neurons. However, in order to encode/decode multiple patterns, these connections could be learned through long-term plasticity such that different memories would be stored in different assemblies, connecting to different readout neurons. Recall could then be implemented by direct stimulation of assembly neurons (Vogels, Sprekeler et al. 2011) or by disinhibition (Barron et al. 2016). In this context, STP could provide a signal that scales with the strength of the memory and that could be used even when the memory is silent and not being recalled.

We have added a paragraph in Discussion where we discuss this in more detail (Lines 332-343).

Minor points:

** line 75: presumably CV means the coefficient of variation, but the acronym should be defined explicitly when first used*

We have defined CV on its first appearance (Line 78).

** Fig 1G: the 1Hz and 8 Hz lines have very similar colours making it difficult to tell which graph is which. I presume the straighter graph is the 1Hz one.*

We have changed the line colors to make it easier to distinguish them.

** line 141: "raster" rather than "rater"*

We have corrected the text.

** line 324 and 327: it is said that the external synaptic weight W_ext = J/3 did not change across simulations while the synaptic weight W_E->E = J did vary. This is confusing as it sounds like W_ext = W_E->E / 3 which both changes and does not change. While we can probably guess what the authors actually mean, the authors should clarify.*

We have made the text clearer (Lines 428-432).

Reviewer #2:

Gallinaro and Clopath report on a model for silent cell assemblies that are under strict excitatory and inhibitory control and encode data in the irregularity of their firing pattern. The authors demonstrate how these cell assemblies could store and relay information to downstream cells. Most striking is the ability for the assemblies to be self-reinforced through spontaneous activity resulting in greater memory longevity. This work: 1) shows how a solitary cell under inhibitory input via iSTDP will increase the irregularity of its firing rate as excitatory input increases. 2) The authors note that in a simulation with multiple input cells and an output neuron, the effect of CV only minimally influenced the firing rate of the output cell. 3) The addition of synaptic facilitation more efficiently modulates the output neuron

firing rate in accordance with the input CV. Importantly this model demonstrates that output cells can interpret the firing irregularity of input cell with the help of STF. 4) Silent assemblies contain traces of previous neuronal activity and this is detectable by an output neuron. The authors suggest that this model could passively accessed for downstream processing. 5) Higher CV (and CC) as found in EI assemblies confers a slower rate of decay on the assembly.

Overall, the authors present an elegant model for how various modes of plasticity contribute to a functional silent assembly. Additionally, information as encoded by the irregularity of the firing rate is particularly intriguing. This mechanism of delayed decay within the network offers one viable explanation for how memories may be preserved. Thus, the paper provides valuable information for the composition of silent cell assemblies and a thought-provoking interpretation of how this model could be applied to our understanding of memory storage, which merit publication. However, I have a few reservations about the study that should be addressed before publication.

Major Points.

While I think the authors present an interesting model for how information could be stored and recalled from silent assemblies. I'm not entirely convinced that the authors address the question of how the these assemblies are formed organically as opposed to hardwiring the connections manually (lines 118 – 120). Rather they demonstrate the critical mechanisms of plasticity that enable the assembly to function once established. This issue should be addressed in the writing.

That's an important point. We have simulated 2 scenarios. Scenario 1: The assemblies are formed by hardwiring connections manually (Supplementary Figure 3). Scenario 2 (Figure 3 from the main text), the assemblies are not formed by hardwiring, but by increasing the external stimulation current to the assembly neurons (Lines 149-150). In that case, both excitatory and inhibitory plasticity are triggered by the increase in firing rate of assembly neurons, leading to the formation of the assembly. Other than driving assembly formation by an increase in external stimulation, other options for forming assemblies "organically" would include increased correlation in the input to assembly neurons (Ocker and Doiron, 2019) and spontaneous formation of assemblies (Montangie et al, 2020), which could be interesting options to explore in future work. According to the reviewer's suggestion, we have added this to the discussion in the main text (Lines 250-258).

Lines 244-248. While it wouldn't be impossible for a biological system to encode data simplistically, the authors should acknowledge that the data could be multiplexed in another way. If each physical channel conveyed one stream of data we are limited to two streams of data but a single physical channel can convey an infinite number of streams (and two physical channels could be used to multiplex independently or in concert.) Modern internet protocols such as HTTP3 are designed to multiplex a large number of streams over a single connection.

The reviewer is right that a more complicated system would be required for multiplexing a large number of streams. The idea of multiplexing two separate streams of information was motivated by the question of how neurons process both bottom-up sensory features and more complex top-down inputs simultaneously in hierarchical architectures, as studied in Naud and Sprekeler, 2018. We

have changed the text to better motivate the multiplexing of two streams of information and to make it clear that a different system would be necessary to multiplex more than two streams (Lines 314-330).

Other.

Line 13, 46, 49, 52, and others. The word "readout" is used throughout the manuscript but it is not always clear whether the authors intend this to be a noun "readout" or a verb "read out".

We have corrected the use of noun "readout" vs verb "read out" in the text.

Line 17. Missing word, "ensembles of neurons is thought to promote".

We have corrected the text.

Line 51. Word order should be adjusted to "EI assembly indeed fire more irregularly".

We have corrected the text.

Line 68. Fixed weight equation, is this a standard notation? $WE1 \rightarrow E2 = J$ seems more intuitive.

It makes sense since this weight refers to a feedforward setup. We have changed the notation relating to Figure 1 to $WE1 \rightarrow E2=J$.

Line 75. Word order should be adjusted, "the neuron always fired at the target rate".

We have corrected the text.

Line 75. CV is first mentioned here but not defined until much later. Please define acronyms at their first mention.

We have defined CV on its first appearance (Line 78).

Figure 1G. The 1 Hz and 8 Hz line colors are nearly indistinguishable. Please change one to provide greater contrast.

We have changed the line colors to make it easier to distinguish them.

Line 143-144. Line 168 implies that Wmax is configurable, so how was it determined in this case?

We have included a rationale for the choice of Wmax in the main text (Lines 174-179)

Line 202. “within assembly” should be “within-assembly” if it is intended to be an adjective.

We have corrected the text.

Line 279-281. Are these standard values? Please provide references.

Yes, these are standard values. For our simulations, the important quantity is the difference between reset and threshold, which is 10mV in our case. Our results would be equivalent to having a threshold of -50mV and a reset of -60mV, which are standard values from experiments. We have added a reference in the Methods section (Lines 374-375).

Line 291-292. Why was this Wmax chosen?

The maximum value of Wmax for the inhibitory-to-excitatory weights was only set to avoid unbound growth of the weights. During the simulations, however, this value was never reached.

Line 333. What is the justification for basing calculations on the last 50 s of the simulation?

We used 50s of simulation in order to have enough inter-spike intervals for a good estimation of the CV.

Line 370-372. How many times were these simulations run?

The network simulations were run 5 times with different random seeds. We have added this information on the Methods section (Line 488).

Sincerely,

Julia Gallinaro and Claudia Clopath
