# Supplementary material for: Memories in a network with excitatory and inhibitory plasticity are encoded in the spiking irregularity
Source: PLoS Comput Biol. 2021 Nov 11;17(11):e1009593. doi: 10.1371/journal.pcbi.1009593 (PMC8610285; doi:10.1371/journal.pcbi.1009593)
Supplement: Attachment — Submitted filename: Gallinaro et al Review.docx [file pcbi.1009593.s010.docx]

Gallinaro and Clopath report on a model for silent cell assemblies that are under strict excitatory and inhibitory control and encode data in the irregularity of their firing pattern. The authors demonstrate how these cell assemblies could store and relay information to downstream cells. Most striking is the ability for the assemblies to be self-reinforced through spontaneous activity resulting in greater memory longevity. This work: 1) shows how a solitary cell under inhibitory input via iSTDP will increase the irregularity of its firing rate as excitatory input increases. 2) The authors note that in a simulation with multiple input cells and an output neuron, the effect of CV only minimally influenced the firing rate of the output cell. 3) The addition of synaptic facilitation more efficiently modulates the output neuron firing rate in accordance with the input CV. Importantly this model demonstrates that output cells can interpret the firing irregularity of input cell with the help of STF. 4) Silent assemblies contain traces of previous neuronal activity and this is detectable by an output neuron. The authors suggest that this model could passively accessed for downstream processing. 5) Higher CV (and CC) as found in EI assemblies confers a slower rate of decay on the assembly.

Overall, the authors present an elegant model for how various modes of plasticity contribute to a functional silent assembly. Additionally, information as encoded by the irregularity of the firing rate is particularly intriguing. This mechanism of delayed decay within the network offers one viable explanation for how memories may be preserved. Thus, the paper provides valuable information for the composition of silent cell assemblies and a thought-provoking interpretation of how this model could be applied to our understanding of memory storage, which merit publication. However, I have a few reservations about the study that should be addressed before publication.

**Major Points**.

While I think the authors present an interesting model for how information could be stored and recalled from silent assemblies. I’m not entirely convinced that the authors address the question of how the these assemblies are formed organically as opposed to hardwiring the connections manually (lines 118 – 120). Rather they demonstrate the critical mechanisms of plasticity that enable the assembly to function once established. This issue should be addressed in the writing.

Lines 244-248. While it wouldn’t be impossible for a biological system to encode data simplistically, the authors should acknowledge that the data could be multiplexed in another way. If each physical channel conveyed one stream of data we are limited to two streams of data but a single physical channel can convey an infinite number of streams (and two physical channels could be used to multiplex independently or in concert.) Modern internet protocols such as HTTP3 are designed to multiplex a large number of streams over a single connection.

**Other**.

Line 13, 46, 49, 52, and others. The word “readout” is used throughout the manuscript but it is not always clear whether the authors intend this to be a noun “readout” or a verb “read out”.

Line 17. Missing word, “ensembles of neurons is thought to promote”.

Line 51. Word order should be adjusted to “EI assembly indeed fire more irregularly”.

Line 68. Fixed weight equation, is this a standard notation? W_E1→E2_ = J seems more intuitive.

Line 75. Word order should be adjusted, “the neuron always fired at the target rate”.

Line 75. CV is first mentioned here but not defined until much later. Please define acronyms at their first mention.

Figure 1G. The 1 Hz and 8 Hz line colors are nearly indistinguishable. Please change one to provide greater contrast.

Line 143-144. Line 168 implies that W^max^ is configurable, so how was it determined in this case?

Line 202. “within assembly” should be “within-assembly” if it is intended to be an adjective.

Line 279-281. Are these standard values? Please provide references.

Line 291-292. Why was this W^max^ chosen?

Line 333. What is the justification for basing calculations on the last 50 s of the simulation?

Line 370-372. How many times were these simulations run?
